# An End-to-End Design and Simulation Methodology for Evaluating Package-Induced Signal Integrity Degradation in PCIe Channels

**DOI:** 10.3390/mi17020218

**Published:** 2026-02-06

**Authors:** Siwook Park, Uichan Kim, Jonghyun Lee, Jiwoon Moon, Yuchul Jung, Youngwoo Kim

**Affiliations:** Department of Semiconductor System Engineering, Sejong University, Seoul 05006, Republic of Korea; princeps015@sju.ac.kr (S.P.);

**Keywords:** end-to-end, package-induced degradation, PCIe, signal integrity (SI)

## Abstract

This paper presents an end-to-end simulation methodology for evaluating package-induced signal integrity (SI) degradation in a peripheral component interconnect express (PCIe) 5.0 channel. By integrating package, printed circuit board (PCB), and add-in card (AIC) structures into a unified simulation flow, the proposed approach enables accurate assessment of system-level eye diagram degradation. Various package-level degradation factors, such as impedance mismatch, meander routing, and via stubs, are assumed and designed to analyze their individual and combined effects on insertion loss, intra-pair skew, and eye diagrams. Results show that even localized discontinuities inside the package propagate and compound through the end-to-end channel, causing a significant reduction in the eye diagram at the system level. These findings demonstrate that package-induced impairments cannot be evaluated solely at the package level but must instead be analyzed within a complete end-to-end channel environment. The proposed methodology provides a practical framework for predicting system-level SI degradation caused by package design choices, offering valuable insights for next-generation high-speed package and channel co-design.

## 1. Introduction

Recent data-centric computing workloads spanning artificial intelligence (AI), machine learning, cloud services, and high-performance computing are driving an insatiable demand for higher data throughput and lower latency in data centers. The increasing demands of AI workloads and data centers drive the need for high-speed, high-bandwidth interconnects. Modern AI accelerators and big-data applications process enormous datasets in parallel, which requires high-speed communication channels between processors, accelerators, memory, and storage. A prime example is the server interconnect, where Ethernet interface speeds have leaped from 100 GbE toward 400 GbE, pressuring input/output (I/O) interfaces to keep pace [1]. Peripheral component interconnect express (PCIe) plays a significant role in this environment by enabling scalable, point-to-point communication between CPUs, GPUs, memory, and accelerators via high-speed serial links. PCIe is a high-performance, general-purpose I/O interconnect defined for a wide variety of future computing and communication platforms [2]. Modern data center architectures rely heavily on PCIe-based interconnect topologies to support high-throughput communication among CPUs, GPUs, accelerators, and storage devices. The PCIe standard has evolved through successive generations to double its per-lane data rate with each iteration. PCIe has become a key high-speed interface in these systems, providing point-to-point, scalable connectivity between CPUs, GPUs, FPGAs, solid-state drives (SSDs), and other devices. Notably, PCIe 5.0 doubled the lane rate to 32.0 GT/s from its 16.0 GT/s predecessor, yielding up to 64 GB/s per × 16 link [3]. However, as PCIe speed increases, maintaining signal integrity (SI) becomes increasingly challenging. Each new generation pushes the physical channel closer to its bandwidth limits, reducing tolerances for signal distortion and jitter. With each successive generation, PCIe operates at higher frequencies where the channel’s voltage and timing margins are drastically reduced, leaving almost no room for error [4]. Accordingly, preserving SI across these high-speed interconnects has become a critical design concern.

**Figure 1:**
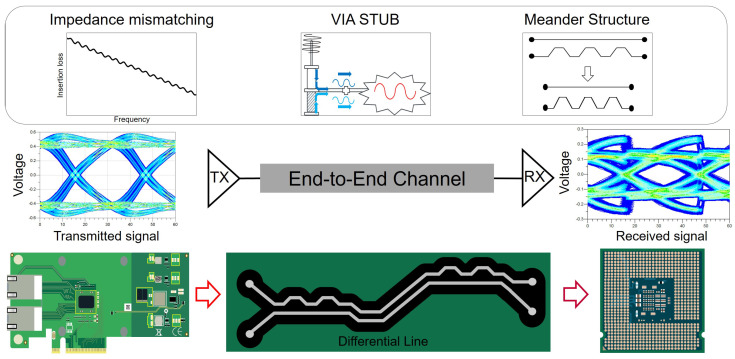
Conceptual overview of package-induced SI degradation mechanisms and their propagation through an end-to-end PCIe channel. Representative package-level impairments, including impedance mismatch, via stubs and meander routing, are illustrated together with their impact on transmitted and received eye diagrams in a system-level channel.

The high-speed channel contains various factors that cause SI degradation [5,6,7,8,9]. Return loss, crosstalk, quarter-wave resonance, and mode conversion are well-known factors that degrade the SI of the high-speed channel. In the case of PCIe channels, several physical discontinuities stand out as particularly critical sources of signal degradation. As illustrated in Figure 1, high-speed PCIe links traverse a complex path including the transmitter package, printed circuit board (PCB) traces, vias, connectors, and the receiver package [3]. Along this path, impedance discontinuity or mismatch can reflect and attenuate the signal, leading to eye diagram closure and degraded timing margins. Likewise, meander routing can add parasitic coupling and intra-pair skew if not carefully designed, further deteriorating the signal. Via stubs are equally problematic; a stub as short as a few tens of mils can form a resonant circuit at high frequencies, reflecting energy back and collapsing the eye diagram opening [10]. These SI degradation factors become more pronounced with each PCIe generation. Yet, comprehensive studies focusing on such effects in advanced package environments or at bleeding-edge speeds are relatively scarce. This lack of detailed analysis is concerning, as these discontinuities are expected to become major bottlenecks for future high-speed interfaces. Indeed, upcoming PCIe generations will tolerate virtually zero excess discontinuity or noise. This trend underlines the need for thorough SI evaluation targeting these three potential sources of signal degradation in high-speed PCIe links.

SI degradation in high-speed serial links has been widely studied, particularly as server systems increase in complexity and component density. It has been shown that PCIe 3.0 channels operating at 8 GT/s can meet insertion loss budgets using standard PCB materials, but 4.0 at 16 GT/s and 5.0 at 32 GT/s often exceed allowable loss limits unless mitigated with low-loss materials or retimers [11,12]. This dramatic reduction in margin with each generation highlights the growing importance of managing SI across the full interconnect. In particular, physical discontinuities such as serpentine routing, via stubs, and impedance mismatches have been identified as major contributors to SI degradation [10,13,14]. Meander delay lines used for length matching introduce intra-pair crosstalk and data-dependent jitter, which reduce eye diagram openings and impair timing margins [13,14]. Similarly, via stubs form resonant structures that degrade return loss and induce inter-symbol interference, especially at frequencies relevant to 4.0 and 5.0 operation [10]. While these findings provide insight into individual discontinuities, they are typically based on localized analysis and do not fully capture the combined effects across a complete system. As PCIe channels often span multiple interconnect elements such as packages, vias, connectors, and multilayer boards, there is a clear need for analysis methodologies that consider the entire signal path. This motivates more comprehensive investigations that go beyond isolated structures and address the broader system-level implications of SI degradation in next-generation PCIe environments.

More recent studies have attempted broader analyses in PCIe channels, but often remain constrained either to the PCB or package domain. They focus on the impact of via stubs in the frequency domain or on package-level impedance mismatches and dense routing-induced crosstalk through eye diagram simulations, revealing a significant reduction in the eye diagram opening [15,16]. However, these analyses are limited to modeling at a single level and fail to capture how such degradations propagate across the complete PCIe channel, including connectors and endpoint devices. Another study simulates end-to-end HDMI channels using input/output buffer information specification-algorithmic modeling interface models (IBIS-AMI), demonstrating the role of equalization and interconnect loss in shaping receiver-side signal quality [17]. However, due to differences in signaling schemes and bandwidth requirements, the findings are not directly applicable to PCIe channels. Furthermore, for earlier-generation PCIe, several studies have explored end-to-end system-level SI performance [18]. However, while the studies are comprehensive, they are tailored to earlier-generation environments and do not fit the tighter loss budgets and increased complexity of modern PCIe links. These gaps underline the need for an end-to-end SI analysis methodology that fully integrates package, PCB, and system-level components to ensure reliable operation at 32 GT/s and beyond. Unlike prior studies that focus either on isolated package structures or non-PCIe interfaces, this work integrates realistic package-induced impairments—namely impedance mismatch, meander routing, and via stubs—into a standardized PCIe 5.0–compliant end-to-end simulation framework based on IBIS-AMI models. The key contribution of this study lies in systematically quantifying how localized package-level signal integrity degradations propagate through subsequent channel segments and compound at the system level, ultimately impacting receiver-side eye metrics. By embedding practical package discontinuities within a full PCIe 5.0 end-to-end channel environment, this work enables a fair and physically realistic evaluation of package design choices that cannot be captured through package-level or partial-channel analyses alone.

In this article, we propose an end-to-end design and simulation methodology for evaluating package-induced SI degradation in PCIe channels to enable accurate system-level analysis and guide practical design optimization. Unlike conventional approaches that analyze the package, PCB, or connectors separately, the proposed method performs a holistic SI evaluation encompassing the full channel from the transmitter die pad, through the package, PCB traces, vias, and connectors, to the receiver die pad. A comprehensive channel model is constructed to include frequency-dependent loss, impedance discontinuities, and crosstalk across all interconnect segments. Based on this model, frequency-domain (S-parameter-based) and time-domain simulations (using IBIS-AMI receiver models) are carried out to assess key metrics such as insertion loss and eye diagrams. By comparing simulation results between channels with and without package-induced impairments, the proposed methodology enables quantitative system-level evaluation of signal degradation originating from the package. This system-level insight assists designers in identifying critical discontinuities and guides them in meeting stringent SI requirements in high-speed channels. The proposed framework provides a practical and effective means to detect and mitigate package-induced SI degradation early in the design process, thereby ensuring sufficient system margin in high-speed PCIe links.

The proposed methodology is validated through application to a practical PCIe 5.0 channel design. A simulation is conducted using a server-grade SSD add-in card, where the end-to-end channel from the host CPU package through the connector to the SSD controller package is fully modeled and analyzed. Based on the actual design layout, package-induced SI degradation is identified, and its impact is evaluated at the system level. This demonstrates that the proposed methodology successfully extends localized package impairments into a comprehensive end-to-end analysis, enabling precise identification and analysis of critical degradation sources. This methodology highlights the necessity of system-level design in managing SI in high-speed PCIe implementations. As interface standards evolve toward higher data rates, including the upcoming PCIe 6.0 specification operating at 64 GT/s with pulse amplitude modulation, design margins continue to shrink while equalization complexity increases. In this context, a robust end-to-end methodology becomes essential for ensuring SI across next-generation interconnects.

## 2. Analysis of Package-Induced Signal Integrity Degradation in PCIe Channels

During PCIe signal propagation, high-speed differential signals travel from the transmitter chip through the complex structures of the semiconductor package, across the package–board interface, and ultimately toward the connector and endpoint device. Notably, the package itself contains several inherent physical discontinuities that can introduce significant SI degradation, particularly at PCIe 5.0 data rates where operating frequencies extend into the tens-of-gigahertz range. Modern packages employ high-density interconnect (HDI) structures, fine-pitch vias, and multi-layer redistribution layers (RDLs). While these features enable compact routing and increased I/O density, they also pose SI challenges arising from geometric irregularities, impedance variations, and resonant behaviors. Accordingly, the primary degradation mechanisms observed in PCIe packages can be broadly classified into three categories: impedance mismatch, meander-induced delay variations, and stub effects. These mechanisms commonly arise in practical package routing and stack-up design and directly impact insertion loss, reflections, and timing skew even in isolated differential pairs. Other effects such as crosstalk and power delivery network (PDN)-induced noise, while also important, predominantly involve multi-lane interactions or signal–power coupling and therefore require different modeling assumptions and analysis frameworks beyond the scope of this study.

### 2.1. SI Degradation Due to Impedance Mismatch

Impedance mismatch is one of the most fundamental sources of signal degradation in high-speed serial links. When the characteristic impedance of a transmission line changes abruptly along the propagation path, a portion of the incident signal is reflected back toward the transmitter. These reflections interfere with the forward-propagating wave, causing amplitude distortion, eye diagram closure, and increased ISI. At PCIe 5.0 data rates, where the channel operates near 32 GT/s with significantly reduced voltage and timing margins, even small impedance deviations of a few ohms can produce measurable degradation in system-level performance. Prior research has established that impedance discontinuities accumulate along the channel and manifest as increased insertion loss and waveform distortion at multi-GHz frequencies [19,20]. When the transmission line impedance differs from that of the transmitter or receiver, part of the transmitted signal is reflected at the discontinuity boundary. These reflected components superimpose with the original waveform, resulting in reduced amplitude, waveform distortion, and timing uncertainty at the receiver. The severity of these reflections increases with both the magnitude of the impedance discontinuity and the frequency content of the transmitted signal, making PCIe 5.0 channels extremely sensitive to even small geometric irregularities.

**Figure 2:**
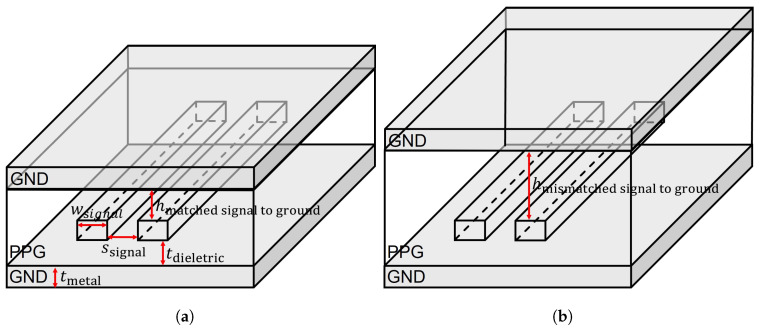
Package structures used to evaluate the impact of impedance discontinuities on signal integrity: (**a**) structure of impedance-matched channel. (**b**) structure of impedance-mismatched channel.

Table 1 and Table 2 summarize the material properties commonly used throughout all simulations. Figure 2 illustrates the package-level differential strip-line structures considered in this study, highlighting the geometric difference between the impedance-matched and impedance-mismatched configurations. These structures are designed to investigate a realistic package-level impedance mismatch scenario commonly encountered in practical semiconductor packages. In an ideal strip-line configuration, the upper and lower reference planes should maintain symmetric dielectric thicknesses to ensure consistent field distribution and stable characteristic impedance. However, in practical package designs, the upper reference plane often contains a power plane rather than a true ground plane, causing the effective return-path layer to shift upward. As a result, the dielectric thickness between the signal trace and its upper reference plane becomes larger than intended, while the lower ground plane remains at the nominal spacing. This asymmetry leads to an imbalance in the electromagnetic field distribution around the differential pair, thereby altering the effective differential impedance. In this study, the mismatched structure is intentionally implemented by increasing the dielectric thickness on the upper reference-plane side from 47 μm to 94 μm, while keeping the lower ground spacing unchanged. This structural modification increases the distance between the signal and reference ground, resulting in significant impedance deviation relative to the matched structure. Such asymmetry is known to cause mode conversion, increased reflections, and differential-to-common-mode conversion phenomena that directly degrade PCIe channel performance at high frequencies [21].

To quantify the extent of impedance deviation caused by the mismatched strip-line structure, a 2D quasi-static (Q2D) field simulation is performed to extract the differential characteristic impedance of both the matched and mismatched configurations. The matched differential pair maintains an impedance close to the PCIe 5.0 specification target of 85 Ω, showing a stable value of approximately 84.2 Ω across the frequency range. In contrast, the mismatched configuration exhibits a significantly elevated impedance, reaching approximately 97 Ω. This deviation of more than 10 Ω clearly indicates that even moderate asymmetry in reference-plane spacing can lead to substantial impedance mismatch. Such impedance variation is critical in PCIe 5.0 channels, where tight differential impedance control is required to minimize reflections, mode conversion, and eye diagram degradation.

**Figure 3:**
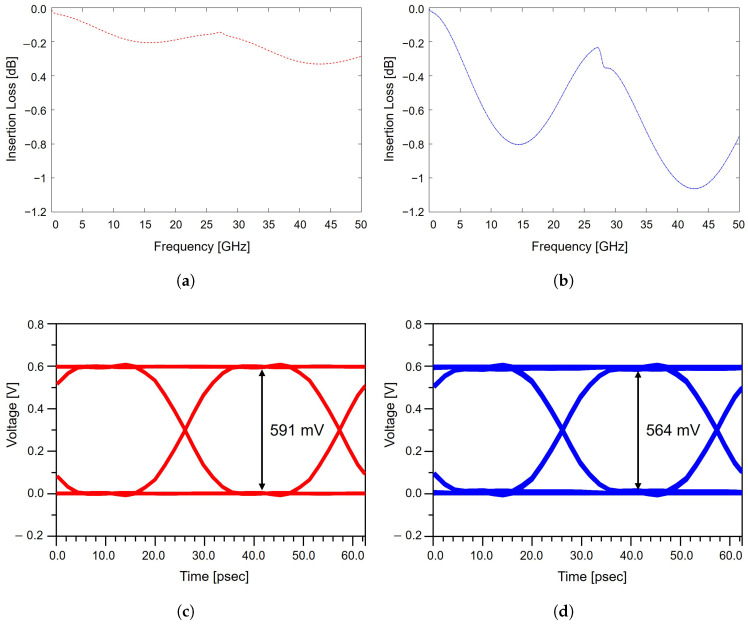
Impact of package-level impedance mismatch on SI: (**a**) insertion loss of an impedance-matched structure. (**b**) insertion loss of an impedance-mismatched structure. (**c**) eye diagram of the impedance-matched channel. (**d**) eye diagram of the impedance-mismatched channel.

The impact of impedance mismatch on PCIe channel performance is evaluated through insertion loss and eye diagram analyses for both matched and mismatched differential strip-line structures. The extracted insertion loss responses are presented in Figure 3a,b. The impedance-matched structure exhibits relatively low attenuation across the PCIe-relevant frequency range, maintaining a smooth and stable loss profile. In contrast, the impedance-mismatched structure shows noticeably higher attenuation, particularly in the mid to high frequency region. This increased loss originates from impedance discontinuities that induce partial reflections of the forward-propagating signal, thereby reducing the effective signal energy delivered to the receiver. The performance degradation associated with impedance mismatch becomes more pronounced when examining the corresponding eye diagrams, as shown in Figure 3c,d. In this analysis, the input voltage is set to 0.6 V, serving as the reference signal amplitude. In the matched case, the eye diagram height reaches approximately 591 mV, corresponding to 98.5% of the input voltage, indicating a well-preserved signal margin. However, the mismatched configuration exhibits a reduced eye diagram height of 564 mV, which corresponds to 94.0% of the input voltage, representing an approximate 4.6% reduction relative to the matched case. This reduction in the eye diagram reflects increased signal distortion and ISI caused by the impedance discontinuity. The narrowed eye diagram opening clearly demonstrates degraded SI and highlights the sensitivity of PCIe 5.0 channels to even moderate impedance variations. These results confirm that deviations from the target differential impedance can lead to measurable degradation in both frequency-domain and time-domain performance. From a design perspective, these results further indicate that careful design of the upper and lower reference planes is critical to maintaining impedance uniformity and minimizing reflection-induced signal degradation in high-speed PCIe packages. As PCIe advances toward higher data rates with increasingly stringent voltage and timing margins, precise impedance control throughout the package and board becomes essential to ensure reliable system-level operation.

### 2.2. SI Degradation Due to Meander Routing

The meander structure is commonly introduced in high-speed differential routing to satisfy strict length-matching requirements between signal pairs. However, the presence of a meander inevitably increases the electrical length of the trace, resulting in greater propagation delay along that segment of the channel. This increased delay can create intra-pair skew when only one line of the differential pair incorporates additional meandering, ultimately degrading the timing alignment at the receiver. In addition to the increased electrical length, the meander geometry introduces a second critical issue that enhances coupling between adjacent segments of the same trace. As serpentine routing places adjacent segments of the transmission line closer together, the electromagnetic fields interact more strongly, leading to additional capacitive and inductive coupling. This effect becomes more pronounced as the spacing between meander segments becomes narrower, which is common in dense routing environments within packages and PCBs. The intensified coupling increases mode conversion and alters the effective characteristic impedance along the meandered section. These local impedance variations cause small reflections and waveform distortions, which accumulate when the signal traverses multiple meander cycles. At high frequencies such as those in PCIe 5.0, even slight phase shifts and coupling-induced perturbations can translate into measurable eye diagram degradation. As a result, although meander routing is often necessary to achieve physical length matching, its adverse impact on SI must be carefully considered, particularly in environments with tight timing and amplitude margins.

To intentionally introduce a controlled length difference within the differential pair, a meander structure is implemented on one of the signal traces. The meander is constructed by adding periodic serpentine segments with a bending angle of $45^{\circ }$, allowing the electrical length to be increased without significantly expanding the lateral routing footprint. This approach provides a practical method for modeling length imbalance that may occur in realistic package routing scenarios, especially in dense interconnect environments where available routing space is limited. The geometric parameters used to implement the meander, such as metal thickness, dielectric thickness, trace width, line spacing, and the total meander length, are summarized in Table 3. These parameters are selected to closely reflect the physical constraints of an actual package stack-up, ensuring that the simulated meander structure captures the coupling and delay characteristics typically observed in high-speed differential interconnects. By applying the meander only to one side of the differential pair, the resulting structure enables systematic evaluation of intra-pair skew and coupling-induced degradation, which are critical considerations for PCIe 5.0.

The timing distortion introduced by the implemented meander structure is evaluated through an intra-pair skew analysis of the differential pair with and without the meander pattern. The reference differential pair maintains identical electrical lengths between the two traces, resulting in no measurable timing offset. In contrast, the meandered structure increases the electrical length of one trace, producing a clear difference in signal propagation delay. The differential pair without the meander exhibits zero skew, whereas the pair incorporating the meander structure shows an intra-pair timing difference of 8 ps between the positive and negative signals. This skew represents the difference in signal arrival times and arises directly from the additional serpentine length introduced on one line. Such intra-pair skew becomes increasingly problematic in high-speed interfaces, where the unit interval (UI) is extremely short, and timing margins are tightly constrained. An 8 ps delay therefore constitutes a non-negligible fraction of the UI, and the resulting misalignment between the differential signals can degrade common-mode rejection, increase jitter, and reduce the eye diagram opening at the receiver. These observations indicate that although meander routing is often necessary for length matching in dense routing environments, its impact on timing integrity must be carefully evaluated to prevent unintended signal degradation in high-speed differential channels.

**Figure 4:**
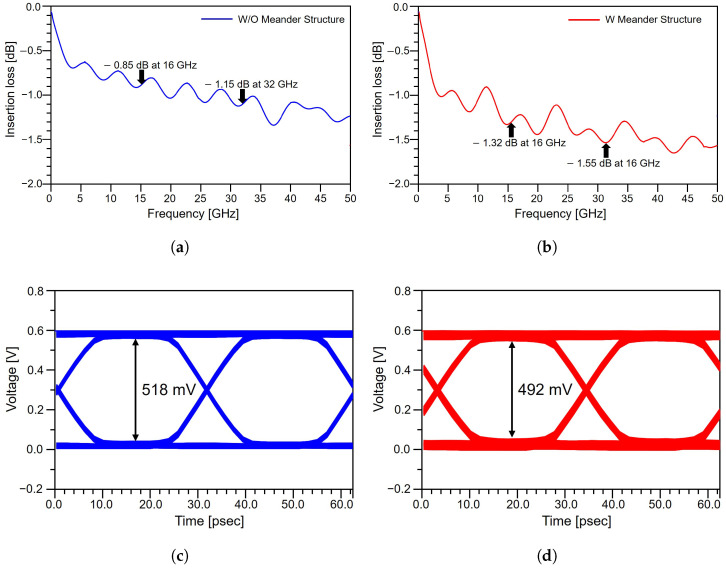
Effect of meander routing on package-level channel performance: (**a**) insertion loss without meander routing. (**b**) insertion loss with meander routing. (**c**) eye diagram without meander routing. (**d**) eye diagram with meander routing.

The impact of the meander structure on signal quality is evaluated through insertion loss and eye diagram analyses of differential lines with and without the meander pattern. The results are presented in Figure 4. The meandered line exhibits noticeably higher insertion loss across the frequency range compared to the non-meandered line. In Figure 4a, the non-meandered line shows an insertion loss of −1.85 dB at the Nyquist frequency for PCIe 5.0. In contrast, as shown in Figure 4b, the meandered line exhibits an insertion loss of −1.32 dB at the Nyquist frequency. This degradation primarily originates from increased electromagnetic coupling between adjacent meander segments, which are routed in close proximity. The enhanced coupling introduces additional parasitic capacitance and inductance, leading to increased attenuation of the high-frequency components of the signal. The corresponding time-domain eye diagrams shown in Figure 4c,d further highlight the adverse effects of the meander geometry. With an input voltage of 0.6 V, the non-meandered line exhibits an eye diagram height of approximately 518 mV, corresponding to 86.3% of the input voltage. In contrast, the meandered line shows a reduced eye diagram height of 492 mV, which corresponds to 82.0% of the input voltage, representing an approximate 5.0% reduction in vertical margin relative to the non-meandered case. This reduction reflects increased waveform distortion and ISI caused by the additional loss and coupling introduced by the meander structure. In addition to amplitude degradation, the meandered line exhibits a noticeable timing shift in the eye diagram. This shift arises from the increased electrical length of the meandered trace, which delays signal propagation relative to the reference line. Such timing displacement can encroach upon, or even exceed, the allowable timing margin in high-speed systems. In PCIe 5.0, where the unit interval is extremely small and timing budgets are tightly constrained, skew-induced timing deviations can directly translate into system-level SI degradation. These results confirm that although meander routing is widely employed for length matching, its use must be carefully controlled in high-speed differential channels. From a practical design perspective, these findings suggest that meander routing should be minimized within the package, where loss budgets are severely constrained, and instead preferentially implemented on the PCB, where comparatively larger loss margins are available.

### 2.3. SI Degradation Due to via Stub

Among the various discontinuities in high-speed interconnects, the via stub is one of the most critical contributors to SI degradation. A via stub refers to the unused portion of a plated-through via that extends beyond the signal transition layer. When a high-speed signal propagates through the via, a portion of the signal energy travels down the stub. Because the stub is an open-ended conductor, the signal that enters the stub is reflected back toward the main signal path, generating a reflected wave with inverted polarity. This reflected wave recombines with the forward-propagating signal, causing amplitude distortion, timing uncertainty, and increased ISI. At multi-GHz frequencies, the via stub behaves as a resonant structure, with its resonant frequency approximately determined by one-quarter wavelength of the stub length. When this resonant frequency approaches the Nyquist frequency of the PCIe channel, the interference between the incident and reflected waves becomes especially pronounced, resulting in deep insertion loss notches and significant eye diagram closure. Prior studies show that even short via stubs can introduce severe attenuation, particularly in PCIe 4.0/5.0 data rates where the channel bandwidth extends beyond 16 GHz [21].

A via stub structure is intentionally implemented beneath the signal via to reproduce a realistic condition commonly encountered in advanced package and PCB designs. In this configuration, the signal via extends beyond the routing layer where the differential pair transitions, leaving an unused residual segment referred to as a via stub. This structure reflects a common industrial scenario in which manufacturing limitations or layer stack-up constraints prevent the complete removal of the unused via portion. In the implemented design, the stub length is set to 395 μm, representing a practical dimension frequently observed in multi-layer package stack-ups. The geometric parameters used to define the via stub structure are summarized in Table 4. These parameters are consistent with typical dimensions found in high-density interconnect packages and provide a realistic representation of how residual via segments contribute to signal reflections and resonance effects in high-speed channels.

**Figure 5:**
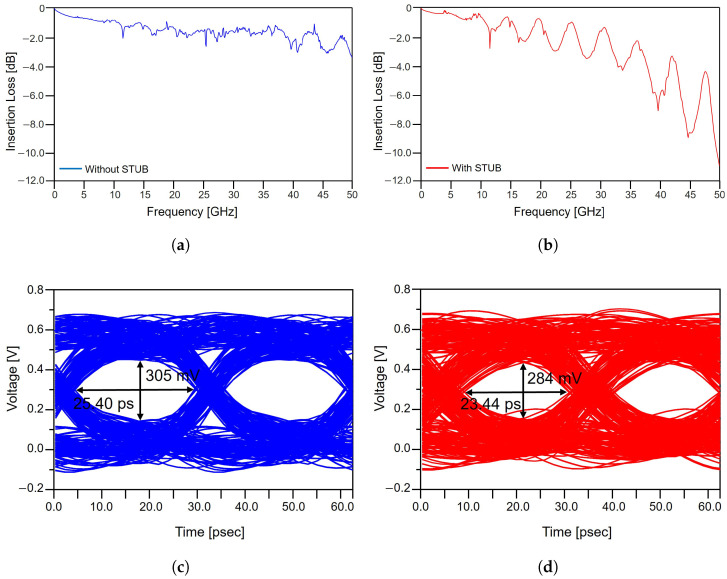
Impact of package-level via stub on signal integrity degradation: (**a**) insertion loss without via stub. (**b**) insertion loss with via stub. (**c**) eye diagram without via stub. (**d**) eye diagram with via stub.

The degradation introduced by the implemented via stub is quantitatively assessed through both frequency-domain and time-domain analyses, as summarized in Figure 5. As shown in Figure 5a, the non-stub channel exhibits a relatively smooth insertion loss profile across the entire frequency range, remaining below −3 dB. In contrast, the channel with a via stub, shown in Figure 5b, demonstrates significantly higher insertion loss over the full frequency range, along with pronounced resonance-induced notches at specific frequencies. This behavior arises because the stub functions as an open-ended transmission-line section, allowing a portion of the incident signal to enter the stub and reflect back toward the main signal path. The superposition of the incident and reflected waves produces frequency-selective loss characteristics, with increasing severity as the stub length grows. When the stub resonance approaches the Nyquist frequency of the PCIe channel, the reflected energy is maximized, leading to deep notches in insertion loss such as those observed between 20 and 40 GHz. At higher frequencies, the reduced wavelength effectively increases the electrical length of the stub, further intensifying the reflection magnitude and exacerbating insertion loss. The adverse impact of the via stub is also evident in the time domain. As shown in Figure 5c,d, the channel without a via stub maintains an eye diagram height of approximately 305 mV and an eye diagram width of 26.40 ps. In contrast, the via-stub configuration exhibits a reduced eye diagram height of 284 mV and a narrower eye diagram width of 23.44 ps, corresponding to a 6.9% reduction in amplitude and an 11.2% reduction in timing margin. The simultaneous degradation of the vertical and horizontal eye diagram margins reflects increased ISI and destructive interference caused by stub-induced reflections. These results confirm that stubs introduce both frequency-selective attenuation and substantial eye closure, making them one of the most detrimental discontinuities in PCIe 5.0 channels. From a package design perspective, these findings underscore the importance of minimizing or eliminating via stubs whenever possible. In advanced package implementations, this can be achieved by employing microvia-based routing to avoid stub formation or by applying backdrilling techniques to remove residual via segments. Such approaches are essential for suppressing resonance effects and preserving sufficient signal margin in high-speed PCIe links.

## 3. Proposal of an End-to-End Method for Quantifying Package Design Impact on System Level Performance

In PCIe 5.0 systems, the end-to-end channel represents the complete signal path extending from the transmitter package to the receiver package. This path includes multiple heterogeneous interconnect segments, specifically the transmitter package, system board, connector, AIC, and the receiver package. The signal first propagates through the transmitter package, traveling through RDLs, solder bumps, and package vias as it exits the chip. It then transitions onto the system board and AIC, where routing traces, vias, and the connector introduce additional channel characteristics, contributing to accumulated insertion loss and reflections. Finally, the signal enters the receiver package, where further impedance variations, via structures, and redistribution layers affect the waveform that reaches the on-die circuitry. Because the end-to-end channel incorporates package segments at both ends, the losses and distortions introduced in each section accumulate along the entire transmission path, collectively influencing the system-level SI. Therefore, adopting a complete end-to-end perspective is essential for accurately assessing PCIe 5.0 channel performance.

**Figure 6:**
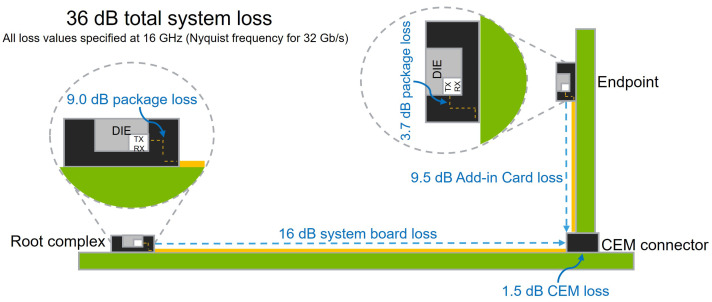
End-to-end PCIe channel loss budget at the Nyquist frequency for 32 GT/s operation. Contributions from the transmitter package, system board, connector, add-in card, and receiver package are illustrated.

Modern PCIe 5.0 links are required to comply with a predefined end-to-end insertion loss budget that is apportioned across the major components of the channel. As summarized in Figure 6, the total allowable channel loss at the Nyquist frequency of 16 GHz is limited to 36 dB and is distributed among the transmitter package, system board, AIC, and connector. Specifically, the PCIe 5.0 specification allocates approximately 9.0 dB to the package, 16.0 dB to the system board, 9.5 dB to the AIC, and 1.5 dB to the card electromechanical (CEM) connector. Because each segment of the end-to-end path consumes a portion of the total loss budget, compliance at the system level cannot be ensured by evaluating only a single component in isolation. Even if the package or board individually meets its assigned budget, the cumulative effects of losses from all channel sections may still violate the overall 36 dB requirement. Furthermore, discontinuities such as impedance mismatch, via stubs, or meander-induced delay can interact with losses in other channel segments and significantly degrade system-level performance. Therefore, a comprehensive end-to-end analysis is essential not only to verify that each component satisfies its individual specification but also to confirm that the combined channel maintains sufficient voltage and timing margin to meet the PCIe 5.0 requirements. This reinforces the necessity of evaluating the full channel as a complete system rather than relying on individual component-level assessments.

The insertion loss characteristics of the system board and the AIC used in this study are obtained from the standard channel models provided by PCI-SIG. These models represent the official compliance structures defined for PCIe 5.0 and are widely used as reference channels for evaluating SI performance in high-speed interconnect systems. Using standardized board models combined with a package model containing practical degradation elements enables a realistic end-to-end evaluation of PCIe 5.0 link performance.

**Figure 7:**
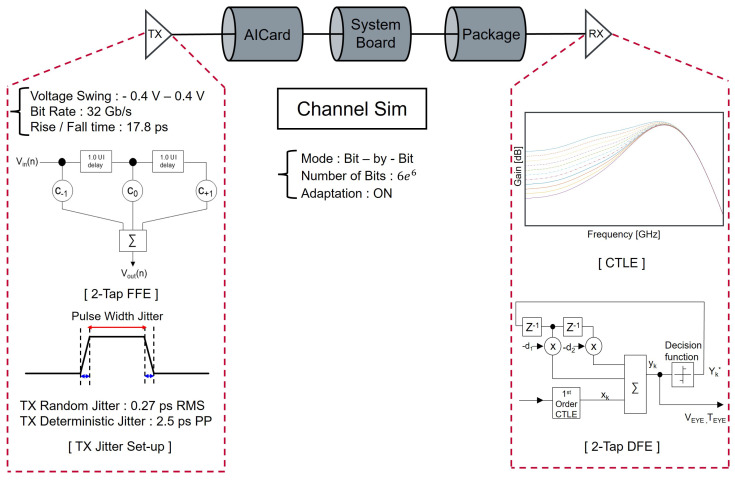
End-to-end PCIe 5.0 simulation configuration. The transmitter FFE, while the receiver utilizes CTLE and DFE to compensate for channel impairments.

For the system-level end-to-end simulation of the PCIe 5.0 channel, the transmitter and receiver equalization parameters are configured according to the PCIe 5.0 Specification. Figure 7 illustrates the overall simulation setup, including the Tx/Rx IBIS-AMI model and the equalization blocks applied to compensate for channel loss. The voltage swing at the transmitter output is set to ±0.4 V, corresponding to the PCIe 5.0 nominal differential swing of 0.8 V. The data rate is configured to 32 GT/s, yielding a Nyquist frequency of 16 GHz. The transmitter rise and fall times are also defined following the specification limits to ensure compliance with the standard. To compensate for channel loss and distortion, a Tx-side feed-forward equalizer (FFE) is applied, while the receiver employs a combination of a continuous-time linear equalizer (CTLE) and a decision feedback equalizer (DFE). In addition, transmitter jitter components such as random jitter and deterministic jitter are included to reflect realistic operating conditions. All simulation parameters are aligned with the PCIe base specification, and the IBIS-AMI models are used to ensure accurate representation of the electrical behavior of the serializer/deserializer (SerDes) blocks. This configuration enables a comprehensive system-level assessment of the end-to-end channel performance under conditions consistent with actual PCIe 5.0 environments. Although the PCIe specification defines allowable ranges for transmitter and receiver equalization parameters, using fixed equalization settings is not sufficient to accurately evaluate system-level signal integrity across channels with different loss and discontinuity characteristics. Each channel configuration investigated in this study exhibits distinct frequency-dependent loss, reflection behavior, and impairment mechanisms arising from variations in package structures. Applying a fixed equalization setting would therefore either over-compensate or under-compensate certain channels, leading to non-representative eye diagram results. To ensure a fair and physically realistic comparison, adaptive equalization is employed so that the receiver dynamically tunes its parameters according to the intrinsic characteristics of each end-to-end channel.

**Figure 8:**
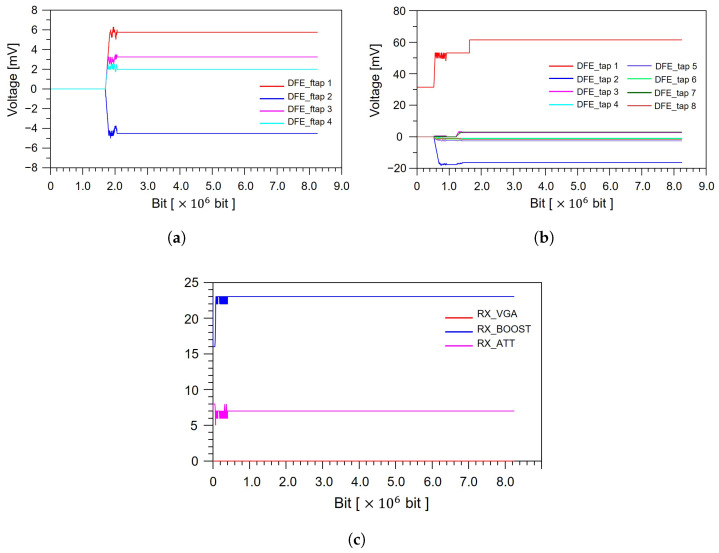
Convergence behavior of receiver equalization during bit-by-bit adaptation: (**a**) evolution of DFE ftap coefficients. (**b**) convergence of 2-tap DFE taps. (**c**) stabilization of receiver VGA, boost, and ATT settings.

In high-speed interfaces such as PCIe 5.0, the channel introduces substantial frequency-dependent loss, reflections, and waveform distortion due to various discontinuities, including package structures, vias, and connector transitions. Because these impairments accumulate along the end-to-end path, adaptive equalization at the receiver is essential to restore the degraded signal and ensure compliance with the required eye diagram margin. Through the adaptation process, the receiver automatically tunes its equalization parameters, such as DFE taps, CTLE boost, and variable gain amplifier (VGA) coefficients, to mitigate ISI and compensate for channel insertion loss. Without such adaptation, the recovered signal would exhibit reduced eye diagram height and width, potentially resulting in performance degradation or link failure under PCIe 5.0 operating conditions. As shown in Figure 8, the equalizer parameters progressively converge during the adaptation process. In the PCIe 5.0 AMI framework, the DFE taps correspond to coefficients that cancel post-cursor ISI components, while the fractional DFE taps (ftaps) provide fine timing adjustments to refine the decision-feedback operation. Together, these mechanisms enable effective ISI mitigation with both coarse and fine resolution. During the initial adaptation phase, the DFE taps and ftaps rapidly adjust to the characteristics of the end-to-end channel and subsequently stabilize once the dominant ISI behavior has been accurately estimated, as illustrated in Figure 8a,b. In parallel, the analog front-end equalization parameters, such as the Rx VGA, CTLE boost, and attenuation (ATT), also converge early in the adaptation sequence as shown in Figure 8c, indicating that the linear equalizer has been properly tuned to the channel conditions. The simultaneous stabilization of both linear (CTLE/VGA) and nonlinear (DFE) equalizers confirms that the receiver dynamically optimizes its parameters to recover the degraded signal to a stable and decodable state. Based on this observed convergence behavior, a total simulation length of $6\times 10^{6}$ bits is employed. In the PCIe 5.0 AMI flow, the receiver equalizer typically reaches saturation after approximately $2\times 10^{6}$ bits, beyond which the DFE taps and CTLE/VGA parameters exhibit negligible variation. However, eye diagrams must be generated using the fully adapted equalizer settings rather than during the transient adaptation phase. Accordingly, an additional $4\times 10^{6}$ bits are simulated after convergence to ensure that eye diagram extraction is performed under steady-state equalized conditions. This approach guarantees that the simulated eye diagram height and eye diagram width accurately represent the true end-to-end channel performance and are consistent with PCIe 5.0 compliance requirements and system-level operating expectations.

## 4. Verification of Package-Induced Impact on End-to-End PCIe Channel

It should be noted that the package-level simulations presented in Section 2 are intentionally designed to isolate and evaluate individual degradation mechanisms under controlled conditions. As a result, signal degradation observed at the package-only level may appear modest. However, in a complete PCIe end-to-end channel, these localized impairments do not remain confined to the package. Instead, they propagate through subsequent channel segments, where they interact with frequency-dependent loss, reflections, and receiver equalization behavior. Consequently, even small amplitude or timing distortions introduced within the package can compound along the channel and lead to disproportionately larger eye closure at the receiver. A PCIe channel forms a complete end-to-end signal path that includes the transmitter IBIS-AMI model, package structure, system board, AIC, and the receiver IBIS-AMI model. Signal propagation through this entire channel involves multiple structural transitions and discontinuities, each of which can introduce significant signal degradation. In particular, the package contains several geometry-dependent features such as impedance mismatches, meander routing, and via stubs that are individually analyzed in Section 2. Although these degradation mechanisms have been previously investigated at the package level, their true impact cannot be fully understood without evaluating how they interact with the system board, connectors, and AIC when forming a complete end-to-end PCIe channel. Even a small discontinuity within the package can propagate along the channel, compounding insertion loss, reflections, and timing distortions as the signal traverses subsequent channel segments. Therefore, the package-induced impairments examined in Section 2 are explicitly incorporated into the end-to-end circuit model to assess their influence on overall system-level PCIe performance. This approach enables direct verification of how localized signal degradation inside the package translates into eye diagram reduction at the receiver, thereby demonstrating the necessity of system-level end-to-end analysis rather than relying on separated package-only evaluations. The system-level degradation trends observed in this section are consistent with previously reported findings in high-speed interconnect and PCIe channel studies. Prior works have shown that impedance mismatches and via stubs induce reflection- and resonance-related insertion loss notches, leading to eye diagram degradation at multi-GHz data rates [5,15]. Similarly, meander routing has been reported to increase electrical length and timing skew, resulting in reduced eye diagram margins in high-speed serial links [13]. Furthermore, the IBIS-AMI-based end-to-end simulation methodology employed in this work follows established modeling approaches adopted in prior full-channel analyses [17]. This consistency with existing literature supports the general validity and credibility of the presented results.

### 4.1. System-Level Impact of Package-Induced Impedance Mismatch

Impedance mismatch inside the package leads to reflections at the discontinuity, which interfere with the forward-propagating signal and cause significant amplitude and timing degradation. Although the differential impedance is designed to meet the 85 Ω PCIe specification, deviations such as the 94 Ω mismatched structure introduce partial reflections that accumulate along the end-to-end channel.

**Figure 9:**
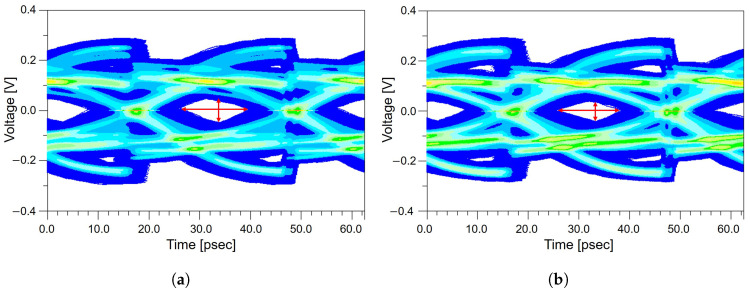
Eye diagram height and width illustrating the effect of package-level impedance discontinuities on system-level signal quality: (**a**) eye diagram of the impedance-matched channel. (**b**) eye diagram of the impedance-mismatched channel, illustrating eye diagram opening degradation caused by package-induced impedance discontinuity and resulting signal reflections.

As shown in Figure 9a, the impedance-matched channel exhibits a wide and well-defined eye diagram opening, indicating stable SI performance. In contrast, the impedance-mismatched channel exhibits a noticeably reduced eye diagram opening compared to the matched case, as shown in Figure 9b. Specifically, the eye diagram height decreases from 49 mV to 39 mV, while the eye diagram width is reduced from 17.81 ps to 16.56 ps, as summarized in Table 5. This degradation is attributed to increased insertion loss, larger residual ISI, and amplified timing uncertainty caused by reflections propagating between the package and board interfaces. Importantly, the impedance mismatch does not remain confined to the local package region but propagates through the system board and AIC, where it compounds with other discontinuities present in the end-to-end channel. As a result, even a localized impedance mismatch within the package can have a measurable and detrimental impact on the entire system-level PCIe channel. From a design perspective, these results highlight the importance of maintaining well-controlled and continuous reference ground planes within the package to preserve impedance uniformity. Proper reference-plane design ensures stable return-current paths and mitigates reflection-induced degradation, enabling robust system-level SI across the complete end-to-end PCIe channel.

### 4.2. System-Level Impact of Meander Routing in Package

Incorporating a meander structure within the package increases the electrical length of the differential pair, causing the signal to experience additional attenuation as it propagates through the end-to-end channel. Because a meandered line is physically longer than a straight trace, the high-frequency components of the signal are more heavily attenuated, resulting in a reduced signal amplitude at the receiver. In addition, the closely spaced segments of the meander geometry introduce parasitic electromagnetic coupling, which distorts the differential waveform and contributes to vertical eye diagram closure at the system level. Another contributing factor is the timing mismatch introduced by the meander structure.

**Figure 10:**
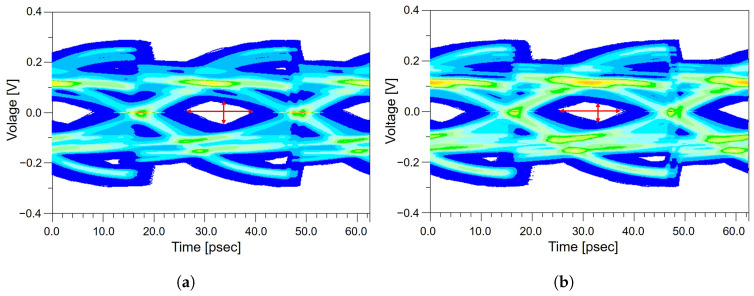
System-level eye diagram height and width comparison of channels with and without the meander routing applied in the package: (**a**) eye diagram of the channel without the meander structure. (**b**) eye diagram of the channel with the meander structure, showing vertical eye opening degradation caused by meander-induced delay and coupling effects.

As analyzed in Section 2, the meander routing generates approximately 8 ps of intra-pair skew. When this skew propagates through the system board, AIC, connectors, and the remainder of the end-to-end channel, it leads to a subtle misalignment in the receiver sampling instant. This misalignment reduces the effective vertical eye diagram margin by shifting the optimal sampling point, causing the eye diagram to appear more compressed even when the horizontal eye diagram opening remains nearly unchanged. These combined effects are clearly observed in Figure 9. As shown in Figure 10a, the channel without the meander structure exhibits a relatively well-defined eye diagram opening, whereas the channel with the meander structure in Figure 10b shows a visibly reduced vertical eye diagram opening. While the eye diagram width of the meandered and non-meandered channels remains nearly identical, the eye diagram height decreases from 49 mV to 44 mV, as summarized in Table 6. This corresponds to a 10.20% reduction in vertical eye diagram opening, demonstrating that even meander routing introduced solely for length matching can negatively impact overall PCIe 5.0 system-level performance. These results further confirm that signal degradation originating within the package does not remain localized, but instead propagates throughout the complete end-to-end channel, ultimately reducing the available eye diagram margin at the receiver. From a design perspective, these observations suggest that meander routing should be minimized within the package, where signal margins are most constrained. Instead, meander structures are more effectively applied in channel segments with greater loss and timing budget, such as the system board, to achieve overall timing alignment while mitigating package-induced signal degradation.

### 4.3. System-Level Validation of Package-Level Routing Optimization

The presence of a via stub in the signal path represents a significant source of signal degradation in high-speed PCIe channels. When a signal propagates through a via transition, a portion of the signal energy enters the unused stub section and is reflected back toward the main transmission path. These reflected components interfere with the forward-propagating signal, introducing amplitude distortion and phase misalignment that accumulate along the end-to-end channel. At high frequencies, the via stub behaves as a resonant transmission-line segment, which exacerbates impedance discontinuities and leads to frequency-dependent reflections. As the signal wavelength becomes comparable to the physical length of the stub, resonance occurs, producing pronounced reflections that attenuate high-frequency signal components and distort the received waveform. Such distortions directly reduce the vertical eye diagram opening and increase residual ISI that cannot be fully mitigated by receiver equalization.

**Figure 11:**
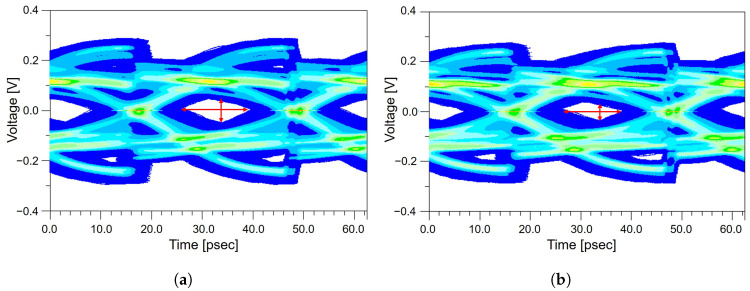
System-level eye diagram height and width comparison of channels with and without a via stub in the package: (**a**) eye diagram of the channel without the via stub. (**b**) eye diagram of the channel with the via stub, illustrating significant eye opening degradation due to stub-induced reflections and resonance effects.

These effects are clearly observed in Figure 10. As shown in Figure 11a, the channel without the via stub exhibits a clear eye opening, whereas the channel with the via stub in Figure 11b shows a severely degraded eye opening due to stub-induced reflections. In the absence of a via stub, the eye diagram maintains an eye diagram height of 49 mV, whereas the introduction of the via stub reduces the eye diagram height to 31 mV, as summarized in Table 7. This corresponds to a 36.73% reduction in the vertical eye diagram opening. The eye diagram width also decreases from 17.81 ps to 16.56 ps, indicating increased timing uncertainty and jitter induced by stub-related reflections. Overall, the results confirm that a via stub constitutes a major discontinuity in the PCIe channel. Although the stub itself exists only within the package region, the distortions it generates do not remain localized but instead propagate through the entire end-to-end channel, ultimately reducing the available eye diagram margin at the system level. From a design perspective, these findings highlight the importance of eliminating via stubs wherever possible by employing microvia-based routing or applying backdrilling techniques to remove residual via segments, thereby preserving SI in high-speed PCIe links.

### 4.4. System-Level Impact of Design Improvement in Package

To ensure reliable operation in high-speed PCIe 5.0 channels, it is essential not only to evaluate the sources of signal degradation but also to investigate whether design improvements applied within the package can yield measurable benefits at the full end-to-end system level. Even small geometric modifications in the package routing can influence insertion loss, impedance continuity, and coupling behavior, and these effects propagate throughout the complete channel. Therefore, system-level evaluation is required to verify the effectiveness of such SI improvement strategies.

**Figure 12:**
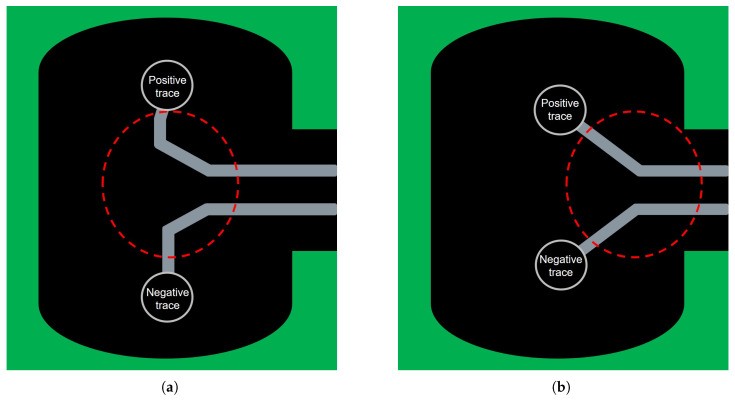
Comparison of package routing geometries near the via region: (**a**) conventional routing with abrupt bends. (**b**) optimized routing employing smoother transitions to mitigate impedance discontinuities.

The routing of the differential pair near the via region is modified to evaluate how localized design improvements within the package influence the overall end-to-end PCIe channel performance. As shown in Figure 12a, in the original layout, the differential traces emerging from the via features two sharp bends, which introduced unnecessary impedance discontinuities and increased coupling imbalance. To alleviate these issues, as shown in Figure 12b, the routing is redesigned to employ a smoother $45^{\circ }$ transition, which not only reduces abrupt changes in trace direction but also satisfies practical design rule constraints related to ground clearance requirements. In practical industrial package and PCB designs, overly smooth or curved routing can violate the minimum spacing to nearby ground features, potentially degrading power integrity or disrupting reference return paths. This geometric refinement helps maintain a more uniform impedance profile around the via, minimizing signal reflections and suppressing excessive coupling variations between the differential lines. By improving continuity in the package-level routing, the modified design is expected to enhance the high-frequency behavior of the signal and reduce distortion as it propagates through the subsequent AIC and system board. The effectiveness of this improvement is verified in the following analysis through a system-level comparison of the resulting eye diagrams.

**Figure 13:**
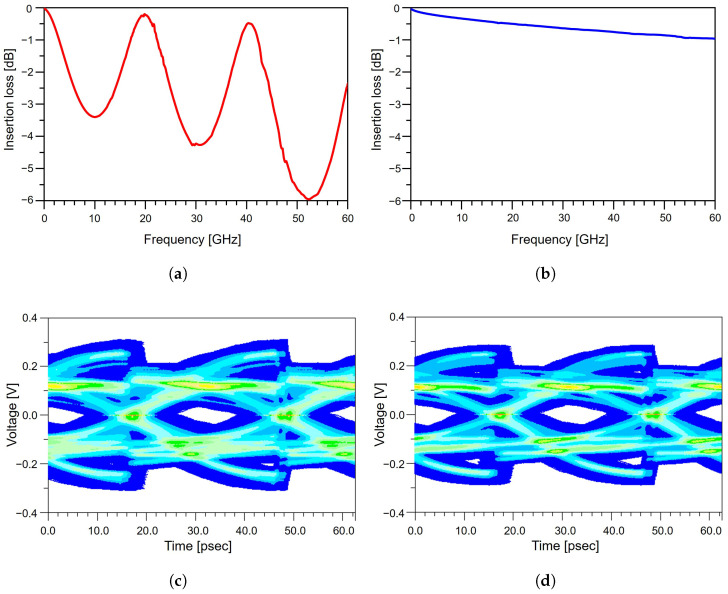
System-level impact of package optimization on signal integrity: (**a**) insertion loss of the baseline package design. (**b**) insertion loss of the optimized package design. (**c**) eye diagram of the baseline design. (**d**) eye diagram of the optimized design.

Figure 13a,b show the frequency-domain comparison of the package-level insertion loss before and after modifying the via trace geometry. The improved routing effectively reduces the high-frequency attenuation that previously resulted from abrupt bends and impedance discontinuities around the via region. As a result, the modified package structure exhibits a noticeably smoother insertion loss profile, especially in the frequency range near the Nyquist frequency of PCIe 5.0, where signal degradation is most critical. To verify whether this local improvement at the package level translates into actual performance benefits at the system level, the modified structure is incorporated into the full end-to-end PCIe channel, and the resulting eye diagrams are analyzed in the time domain. As shown in Figure 13c,d, the improved design produces a significantly taller and wider eye diagram height and width compared to the original structure. The eye diagram height increases from 36 mV to 50 mV, and the eye diagram width expands from 16.25 ps to 18.12 ps, as summarized in Table 8. This enhancement indicates that the reduction in package-level discontinuities not only mitigates insertion loss but also enables the equalizer to more effectively restore the waveform, thereby improving the overall signal margin at the receiver. These results demonstrate that even modest geometric refinements within the package routing can generate meaningful improvements in end-to-end PCIe channel performance. Consequently, design optimization within the package should be evaluated in the broader context of the full system-level channel, as the benefits propagate through the AIC, system board, connectors, and ultimately to the receiver.

## 5. Conclusions

In this article, a comprehensive end-to-end design and simulation methodology was presented to evaluate SI degradation in PCIe 5.0 channels, with a particular emphasis on impairments originating from the package. Unlike conventional approaches that primarily investigate package-induced effects in isolation or analyze separated interconnect structures such as PCB vias or non-PCIe serial links, this work demonstrated that such localized analyses are insufficient for modern high-speed interfaces operating at tens of gigahertz. As shown throughout this study, even minor geometric discontinuities within the package, such as impedance mismatches, meander routing, and via stubs, can propagate through the entire PCIe link and meaningfully degrade the system-level eye diagram. Through detailed frequency-domain and time-domain analyses, this work systematically examined how package-level discontinuities interact with the system board and AIC to compound insertion loss, reflections, ISI, and timing uncertainty. The results clearly indicate that degradation mechanisms originating from the package do not remain locally but instead accumulate along the end-to-end channel, ultimately reducing the available voltage and timing margins at the receiver. Based on the proposed end-to-end methodology, practical design guidelines were also derived to mitigate package-induced SI degradation. The results suggest that precise impedance control through well-maintained reference planes, minimization of meander routing within the package, and elimination of via stubs through microvia or backdrilling are critical to preserving signal margin in high-speed PCIe channels. Overall, the proposed methodology provided a robust framework for accurately evaluating and mitigating SI degradation in next-generation PCIe channels. As interface standards continue to evolve toward higher data rates with increasingly stringent margin requirements, the importance of end-to-end system-level analysis will continue to increase. The approach presented in this work enabled designers to identify critical degradation sources early in the design process and to implement effective mitigation strategies, thereby ensuring reliable operation of high-speed PCIe interconnects in advanced packaging environments.

## Figures and Tables

**Table 1:** Material properties of the designed structure.

	Symbol	Value
Material Properties	$\varepsilon_{\mathrm{dieletric}}$	3.3 at 5.8 GHz
	$\tan\delta_{\mathrm{dieletric}}$	0.0044 at 5.8 GHz

**Table 2:** Physical dimensions used in the package-level impedance mismatched simulations.

	Symbol	Value
Physical Dimensions	$t_{\mathrm{metal}}$	15 μm
	$t_{\mathrm{dieletric}}$	47 μm
	$w_{\mathrm{signal}}$	29 μm
	$s_{\mathrm{signal}}$	63 μm
	$h_{\mathrm{matched}\mathrm{signal}\mathrm{to}\mathrm{ground}}$	47 μm
	$h_{\mathrm{mismatched}\mathrm{signal}\mathrm{to}\mathrm{ground}}$	94 μm

**Table 3:** Physical dimensions used in the package-level meander simulations.

	Symbol	Value
Physical Dimensions	$t_{\mathrm{metal}}$	15 μm
	$t_{\mathrm{dieletric}}$	30 μm
	$w_{\mathrm{signal}}$	29 μm
	$s_{\mathrm{signal}}$	63 μm
	$l_{\mathrm{meander}}$	6.47 mm

**Table 4:** Physical dimensions defining the package-level via stub structure used in the simulations.

	Symbol	Value
Physical Dimensions	$t_{\mathrm{metal}}$	15 μm
	$t_{\mathrm{dieletric}}$	30 μm
	$w_{\mathrm{signal}}$	29 μm
	$s_{\mathrm{signal}}$	63 μm
	$l_{\mathrm{stub}}$	395 μm

**Table 5:** Comparison of eye width and eye height for impedance-matched and impedance-mismatched package structures.

Structure	Eye Width [ps]	Eye Height [mV]
Impedance matched	17.81	49
Impedance mismatched	16.56	39

**Table 6:** Impact of meander routing on system-level eye metrics.

Structure	Eye Width [ps]	Eye Height [mV]
Without meander	17.81	49
With meander	17.81	44

**Table 7:** Comparison of eye width and eye height for channels with and without via stubs.

Structure	Eye Width [ps]	Eye Height [mV]
Without stub	17.81	49
With stub	16.56	31

**Table 8:** Summary of system-level eye metrics for improved package design configurations.

Structure	Eye Width [ps]	Eye Height [mV]
Previous design	16.25	36
Improved design	18.12	50

## Data Availability

The original contributions presented in this study are included in the article. Further inquiries can be directed to the corresponding author.
